# A Microbial Transformation Model for Simulating Mammal Metabolism of Artemisinin

**DOI:** 10.3390/molecules24020315

**Published:** 2019-01-16

**Authors:** Yue Ma, Peng Sun, Yifan Zhao, Kun Wang, Xiaoqiang Chang, Yue Bai, Dong Zhang, Lan Yang

**Affiliations:** 1 Institute of Chinese Materia Medica, China Academy of Chinese Medical Sciences, Beijing 100700, China; yuema_2016@126.com (Y.M.); psun@icmm.ac.cn (P.S.); zyfan_666@163.com (Y.Z.); supervipwk@163.com (K.W.); 19937381620@163.com (X.C.); by15076380819@163.com (Y.B.); 2 Artemisinin Research Center, Beijing 100700, China

**Keywords:** microbial transformation, *Cunninghamella elegans*, metabolite, identification, artemisinin, UPLC-ESI-Q-TOF-MS^E^, UNIFI software, in vivo

## Abstract

Artemisinin (ART) is a highly effective antimalarial agent isolated from the traditional Chinese herb Qinghao. Metabolism of ART and its derivatives in the body is one of the most pressing issues for pharmaceutical scientists. Herein, an efficient in vitro microorganism model for simulation of metabolism of ART in vivo was developed employing *Cunninghamella elegans.* Metabolites in the microbial transformation system and plasma of mice pre-administrated ART orally were analyzed by ultra-performance liquid chromatography (UPLC)-electrospray ionization (ESI)-quadrupole time-of-flight (Q-TOF)-mass spectrometry (MS^E^) combined with UNIFI software. Thirty-two metabolites were identified in vitro and 23 were identified in vivo. After comparison, 16 products were found to be common to both models including monohydroxylated ART, dihydroxylated ART, deoxyartemisinin, hydroxylated deoxyartemisinin, hydroxylated dihydroartemisinin (DHA), and hydroxylated deoxy-DHA. These results revealed that *C. elegans* CICC 40250 functioned as an appropriate model to mimic ART metabolism in vivo. Moreover, an overall description of metabolites of ART from *C. elegans* CICC 40250 has been provided. Notably, DHA was detected and identified as a metabolite of ART in mouse plasma for the first time.

## 1. Introduction

Artemisinin (ART), a well-known natural product, was first isolated from the leaves of *Aremisia annua Linn* by Professor Tu and coworkers in the 1970s [1,2]. Millions of lives threatened by malaria have been saved by this excellent agent in the last decades [3]. Recently, ART was found to exhibit extensive bioactivities such as anti-schistosoma, antitumor, immunosuppression, and antifibrosis [4,5]. Insight of its significant applications in pharmaceuticals and bioactive agents [6] as well as the metabolites of ART in the body are of great interest to researchers.

Due to the rapid and extensive metabolism of ART in the body [7,8,9], direct study of its metabolites in the body is challenging [10,11,12,13,14]. Significant progresses have been made to predict the drug disposal process, and a series of metabolites have been predicted in previous studies [15,16,17,18,19,20]. In spite of these brilliant works, the efficient tools to generate abundant metabolites in vitro are still in demand, which could provide the possibility for further bioevaluation and synergistic effect study of the metabolites.

In recent years, microbial transformations have become powerful routes for metabolic simulations [21], and have been used to study metabolism of a variety of clinical drugs, such as fenofibrate [22], imipramine [23], monensin A [24], and cinobufagin [25]. Compared with in vivo metabolism studies and microsomal methods, microbial transformation is more convenient and cost-effective, especially with the advantage of scalability in vitro. Over the last few decades, a number of ART metabolites were obtained and identified via several species of fungi [26,27], including artemisitone-9 [28], 6β-hydroxyartemisinin [29,30], 7α-hydroxyartemisinin, 7β-hydroxyartemisinin, 7β-hydroxy-9α-artemisinin [31] 1α-hydroxyartemisinin [32], 10β-hydroxyartemisinin, C-9 acetoxy artemisinin [33], 9α-hydroxyartemisinin, deoxyartemisinin [34] 4α-hydroxy-1-deoxyartemisinin [35,36], 5α-hydroxyartemisinin [37], artemisinin G, 6β, 7α-hydroxyartemisinin [38], and 14-hydroxy-deoxyartemisinin [39]. However, whether these compounds were produced in vivo has not been verified.

Herein, to get some insight of ART metabolism and also as parts of our ongoing projects on ART research [40,41], we conducted our study on the metabolism simulation employing of ART employing the fungus *Cunninghamella elegans* CICC 40250. Divergent metabolites were produced via this microbial model in vitro. Moreover, metabolites in mouse plasma were also identified and compared with the products generated in vitro in our study. This study demonstrated the possibility of obtaining potentially valuable metabolites in large quantities, and a convenient and low-cost microbial model for ART metabolism simulation would help for obtainment of new candidates and exploration of the mechanism of action.

## 2. Results

### 2.1. Optimization of Ultra-Performance Liquid Chromatography (UPLC) Conditions

To improve the resolution and intensities of chromatographic peaks, various compositions of mobile phase (methanol, acetonitrile, and formic acid) were tested to optimize chromatographic conditions. As a result, acetonitrile (containing 0.1% formic acid) and water (containing 0.1% formic acid) were used as the mobile phases. The gradient program for samples, which included five segments, was as follows, 5–100% B from 0 to 15 min, 100–5% B from 15 to 17 min, and a post-run of 3 min for column equilibration. The flow rate was 0.4 mL min^−1^, and the temperature was held at 25 °C throughout the period of analysis.

### 2.2. Optimization of Sample Preparation

Protein precipitation (acetonitrile and methanol) and solid-phase extraction (SPE) were evaluated for extraction of metabolites. SPE allowed for simultaneous extraction of all target compounds. Greater amounts of target compounds were obtained using SPE compared with protein precipitation. Moreover, methanol, selected as the elution solvent for SPE, was used at the optimal flow rate of one drop every 3 s. To determine the optimal volume of methanol required to elute target compounds from the SPE cartridge, elution was performed twice with 1.0 mL methanol each. Our results showed that 1.0 mL of methanol was sufficient to elute the target compounds. As a result, the optimize elution program of SPE was described as 1.0 mL of water followed by 1.0 mL of methanol at the flow rate of one drop every 3 s. The target compounds were gathered in the organic phase.

### 2.3. Identification of Reference Substances

**Figure 1:**
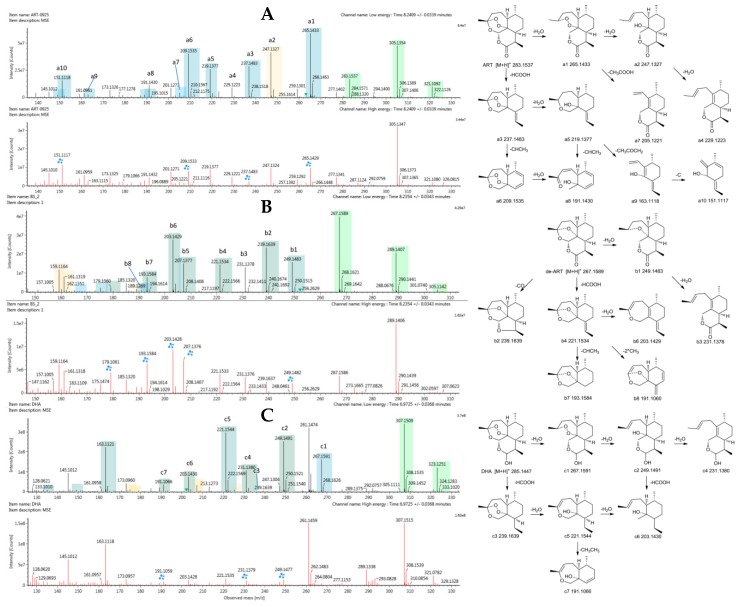
Mass spectrometry (MS^E^) spectra (**A**) artemisinin (ART), (**B**) deoxyartemisinin (de-ART), and (**C**) dihydroartemisinin (DHA) as well as the proposed fragmentation pathways. A blue box behind the low-energy fragment ions shows in-source fragment ions and a green box indicates adduct clusters.

The reference substances ART, deoxyartemisinin (de-ART), and dihydroartemisinin (DHA) were used to establish reference fragmentation patterns. The molecular ions [M + H]^+^, [M + Na]^+^, and [M + K]^+^ of ART in positive mode were observed at *m*/*z* 283.1537, 305.1354, and 321.1092, respectively. The fragment ions at *m*/*z* 265, 247, and 229 were generated by successive loss of a water from *m*/*z* 283, and *m*/*z* 237 also resulted from loss of HCOOH from the fragment represented by *m*/*z* 283. Moreover, the fragment ion at *m*/*z* 219 was generated by loss of a water from the fragment represented by *m*/*z* 237. In addition, the fragment ion *m*/*z* 209 was generated from loss of CHCH_3_ from the *m*/*z* 237 fragment, which was also the mechanism of transition from *m*/*z* 219 to *m*/*z* 191. The fragment represented by *m*/*z* 205 was generated by loss of CH3COOH from the *m*/*z* 265 fragment. Mass spectra and the fragmentation scheme for ART are shown in Figure 1A.

The reference substance de-ART showed similar characteristic product ions to ART. The molecular ions were represented by *m*/*z* 267.1589 ([M + H]^+^), 289.1407 ([M + Na]^+^), and 305.1142 ([M + K]^+^). The fragment ions at *m*/*z* 249, 231, 221, and 203 were 16 Da less than the corresponding fragment ions of ART. In addition, the fragment ion at *m*/*z* 239 was generated from loss of CO from the *m*/*z* 267 fragment. Compared with ART, the fragments of de-ART were less susceptible to loss of water, but more susceptible to loss of CO. Mass spectra and the fragmentation scheme for de-ART are shown in Figure 1B.

DHA showed molecular ions [M + H]^+^, [M + Na]^+^, and [M + K]^+^ at *m*/*z* 285.1447, 307.1509, and 323.1251, respectively. Fragments of DHA were similar to those of ART, with *m*/*z* 267, 249, and 231 resulting from successive loss of water from the *m*/*z* 285 fragment, and *m*/*z* 239 resulted from *m*/*z* 285 by loss of HCOOH. The fragment ions at *m*/*z* 221 and *m*/*z* 203 resulted from loss of one water and two waters, respectively, from the *m*/*z* 239 fragment. Mass spectra and the fragmentation scheme for DHA are shown in Figure 1C.

### 2.4. Identification of Metabolites

**Figure 2:**
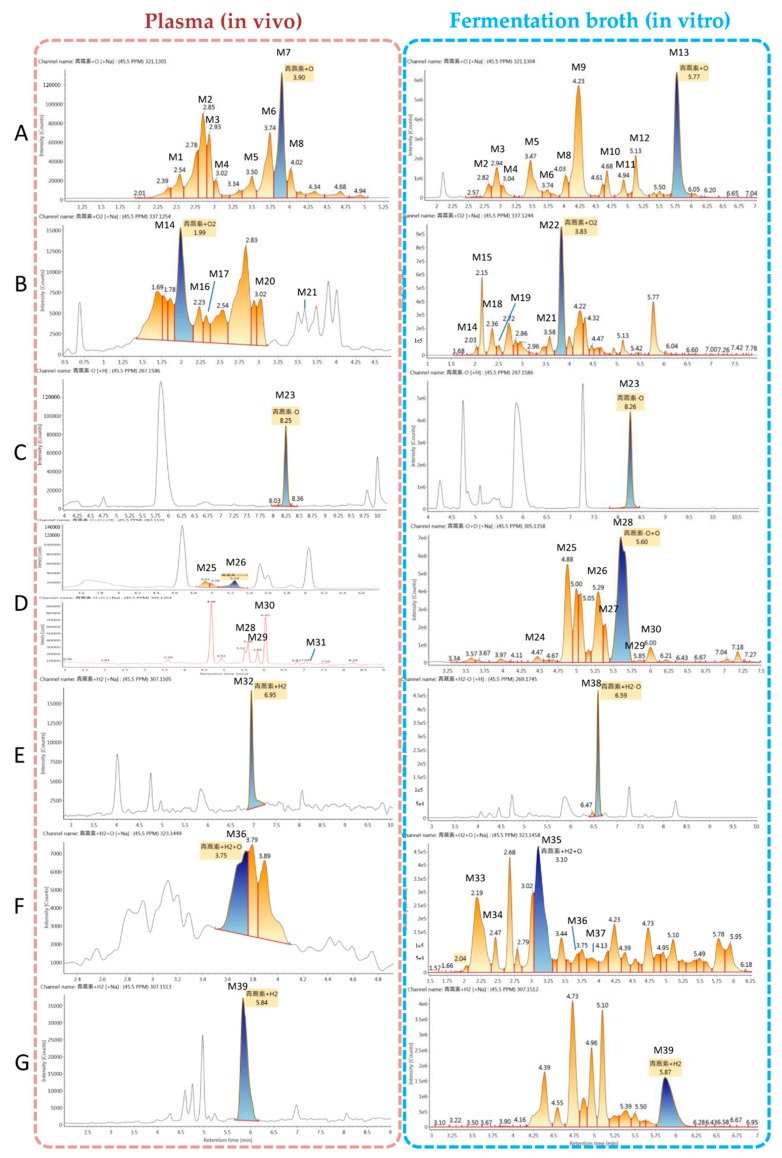
Extracted ion chromatograms of the artemisinin (ART) metabolites obtained from mouse plasma (left) and fermentation broth of *C. elegans* CICC 40250 (right). (**A**) monohydroxylation metabolites (M1–M13), (**B**) dihydroxylation metabolites (M14–M22), (**C**) deoxyartemisinin (de-ART; M23), (**D**) deoxygenation followed by hydroxylation metabolites (M24–M31), (**E**) dihydroartemisinin (DHA; M32) and deoxygenated DHA (M38), (**F**) hydrogenated DHA (M33–M37), and (**G**) hydrogenation followed by deoxidization of DHA (M39).

To evaluate the ability of microbial transformation models to mimic metabolism in vivo, metabolites in mouse plasma and in a microbial transformation system were evaluated by UPLC-electrospray ionization (ESI)-quadrupole time-of-flight (Q-TOF)-mass spectrometry (MS^E^) and identified using UNIFI 1.9 software (Waters, Manchester, UK). Analytes in each sample were compared based on characteristic mass spectrometric behavior, including parent ions, internal cleavage in the ion source, and characteristic fragment ions of each metabolite, as well as by retention time. Compared with the peaks in the corresponding blank sample, a total of 39 additional peaks were observed and presumed to be metabolites in plasma and the microorganism system. The observed metabolites were generated by monohydroxylation and dihydroxylation of ART, de-ART, hydroxy-de-ART, DHA, hydroxy-DHA, and hydroxy-deoxy-DHA. All metabolites identified were listed in Table 1. Extracted ion chromatograms of the metabolites in plasma and fermentation broth are shown in Figure 2.

#### 2.4.1. Structural Elucidation of ART Metabolites

**Figure 3:**
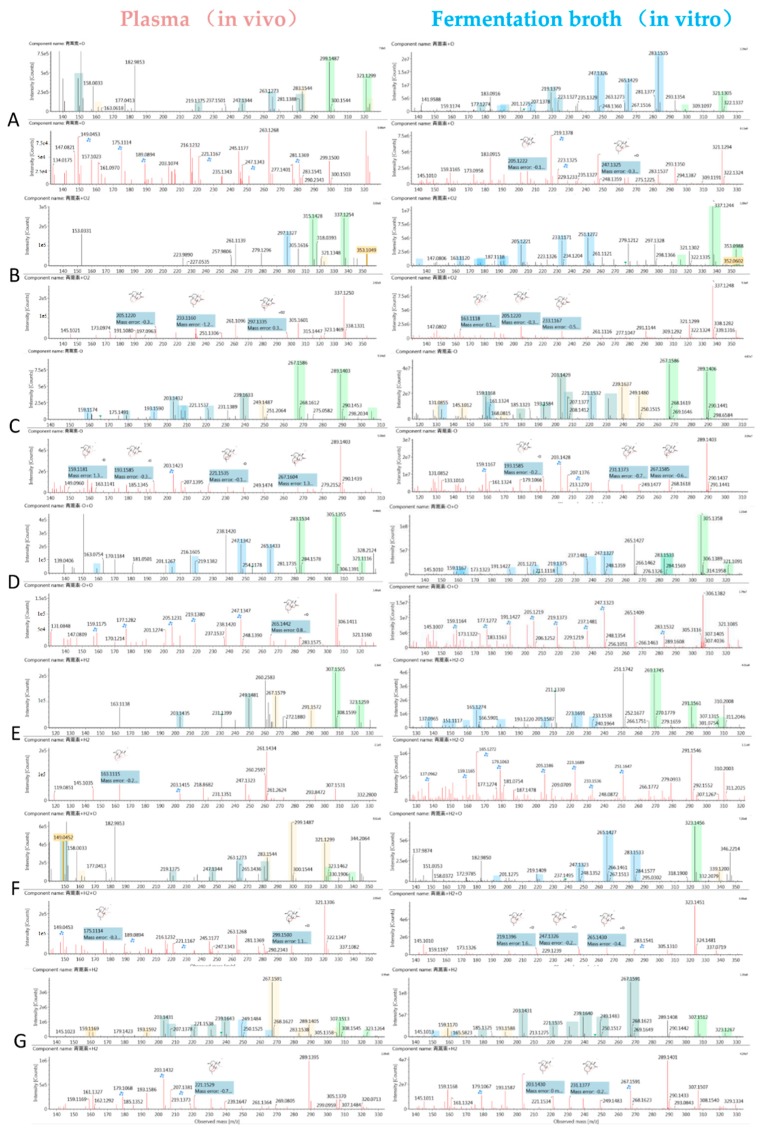
Ultra-performance liquid chromatography-electrospray ionization-quadrupole time-of-flight-mass spectrometry (UPLC-ESI-Q-TOF-MSE) spectra of metabolites in mouse plasma (left) and UPLC-ESI-Q-TOF-MSE spectra of metabolites in microorganisms (right) from the same sample and fragmentation sites in the high energy spectrum. (**A**) Monohydroxylation metabolite, (**B**) dihydroxylation metabolite, (**C**) deoxyartemisinin (de-ART), (**D**) deoxygenation followed by hydroxylation metabolite, (**E**) dihydroartemisinin (DHA) and deoxygenated DHA, (**F**) hydrogenated DHA, and (**G**) hydrogenation followed by deoxidization of DHA. A blue box behind the low-energy fragment ions shows in-source fragment ions and a green box indicates adduct clusters.

All metabolites were detected within a 16 min chromatographic method. Twenty-two metabolites resulted from hydroxylation, with 13 resulting in monohydroxylated derivatives, ART + O, and nine dihydroxylated derivatives, ART + 2O. Monohydroxylated ART (M1–M13) metabolites were detected between 2.6 to 5.8 min, and exhibited molecular ions at *m*/*z* 305 ([M + Na]^+^) and *m*/*z* 299 ([M + H]^+^). They showed similar parent ions to ART except with a 16 Da (an oxygen atom) mass shift, as shown in Figure 3A. A fragment with *m*/*z* 281 resulted from loss of water from the molecular ion at *m*/*z* 299. High-energy spectra showed that the fragments *m*/*z* 263 and *m*/*z* 245 resulted from successive loss of a water from the *m*/*z* 281 fragment. The fragment ion *m*/*z* 253 resulted from loss of HCOOH from the *m*/*z* 299 fragment, and the transition from *m*/*z* 263 to *m*/*z* 217 resulted from the same mechanism. In addition, the fragment ion at *m*/*z* 235 resulted from loss of water from the *m*/*z* 253 fragment. Fragment *m*/*z* 221 resulted from loss of CH_3_COOH from the *m*/*z* 281 fragment.

Metabolites M14–M22 were predicted to be dihydroxylated derivatives, and eluted between 2.0 to 3.8 min. Among these, four metabolites had not been identified in vivo in previous studies. The dihydroxylated ART metabolites showed molecular ions at *m*/*z* 315 ([M + H]^+^) and *m*/*z* 337 ([M + Na]^+^) (Figure 3B). Consistent with monohydroxylation products of ART, they showed a series of product ions resulting from loss of H_2_O, HCOOH, and CH_3_COOH. More fragments resulted from loss of water, demonstrating that dihydroxylated ART metabolites had more hydroxyl groups than the monohydroxylated metabolites.

Deoxyartemisinin (M23, de-ART), a known mammalian metabolite of ART, was identified in both plasma and fermentation broth (Rt = 8.3). De-ART showed molecular ions at *m*/*z* 289 ([M + Na]^+^) and *m*/*z* 267 ([M + H]^+^), and the product ions were consistent with the reference substance (Figure 3C).

Metabolites M24–M31 were proposed to be deoxygenation and hydroxylation metabolites (de-ART + O), which showed molecular ions at *m*/*z* 305 ([M + Na]^+^) and *m*/*z* 283 ([M + H]^+^). The peaks corresponding to these types of metabolites eluted between 4.5 to 7.8 min. These were isomers of ART, and exhibited the same fragments as ART, such as *m*/*z* 265, *m*/*z* 247, *m*/*z* 237, *m*/*z* 219, *m*/*z* 209, and *m*/*z* 191. The fragments *m*/*z* 267, 249, 239, and 221 were from addition of H_2_ to corresponding fragment ions of ART (Figure 3D).

Furthermore, DHA (M32, Rt = 7.0 min) was identified in plasma for the first time. DHA exhibited similar molecular ions and fragment ions as the reference substance (Figure 3E).

Hydroxy-DHA metabolites (DHA + O and M33–M37) were identified in both plasma and in the microorganism model. They showed a molecular ion at *m*/*z* 323 ([M + H]^+^). In high-energy spectra, the fragments *m*/*z* 285 and *m*/*z* 283 were proposed to represent loss of water with or without addition of H_2_. The fragments corresponding to *m*/*z* 267, 249, and 231 resulted from successive loss of water, the same mechanism responsible for *m*/*z* 265, 247, and 229. The fragment *m*/*z* 219 was generated from *m*/*z* 265 following loss of HCOOH, the same mechanism responsible for transition from *m*/*z* 283 to *m*/*z* 237 and from *m*/*z* 285 to *m*/*z* 339 (Figure 3F).

Dihydrodeoxyartemisinin (de-DHA and M38) showed molecular ions at *m*/*z* 291 ([M + Na]^+^) and 269 ([M + H]^+^), with fragment ions at *m*/*z* 251, 233, and 215 proposed to result from successive loss of water from the molecular ion. In addition, the fragment *m*/*z* 223 was generated from *m*/*z* 269 following loss of HCOOH. The fragment *m*/*z* 205 resulted from *m*/*z* 223 after loss of a water (Figure 3D).

Metabolite M39 was predicted to be hydroxy-dihydrodeoxyartemisinin (de-DHA + O), and was detected at 5.8 min. It was an isomer of DHA, with the same molecular ion at *m*/*z* 307 ([M + Na]^+^) and fragment ions at *m*/*z* 267, 249, 231, and 203. In addition, the fragment *m*/*z* 239 was generated from the molecular ion following loss of HCOOH, and the fragment *m*/*z* 221 resulted from loss of water from *m*/*z* 239 (Figure 3G).

#### 2.4.2. Comparison of Results

**Figure 4:**
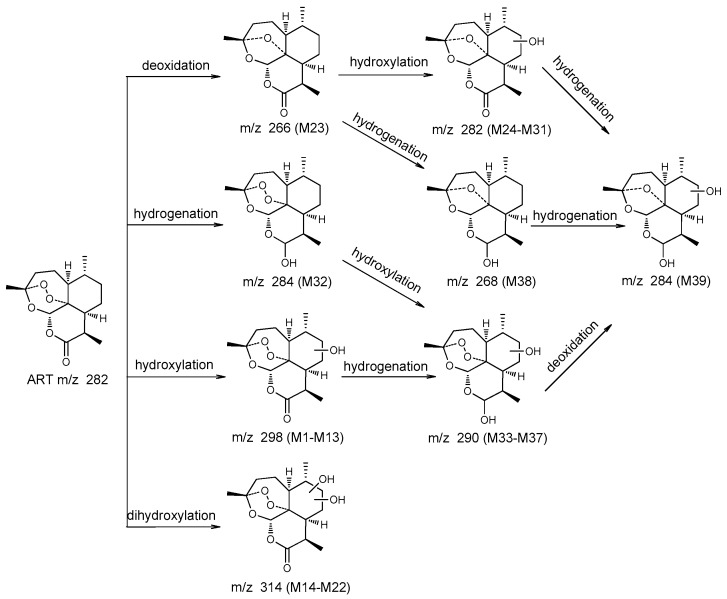
Proposed metabolic pathways of ART.

To evaluate the capacity of *C. elegans* CICC 40250 to produce mammalian metabolites, in vitro results were compared with in vivo results. A total of 23 metabolites and 32 metabolites were identified in mouse plasma and the microbial transformation system, respectively. Among these, 16 metabolites were common to both systems. The metabolites generated were seven monohydroxylated derivatives, two dihydroxylated derivatives, deoxyartemisinin, four hydroxylated de-ART derivatives, one hydroxylated DHA derivative, and one hydroxylated deoxy-DHA derivative. In addition, some metabolites were only detected in mouse plasma, including DHA (M32), two monohydroxylated ART derivatives (M1 and M7), three dihydroxylated ART derivatives (M16, M17, and M20), and hydroxylated de-ART (M29). Similarly, some metabolites were only identified in microbial transformation broth. Compared with the in vivo results, more low polarity metabolites of monohydroxylated and dihydroxylated ART, and hydroxylated DHA were detected only in vitro. The proposed metabolic pathways for ART were shown in Figure 4.

## 3. Discussion

The metabolism simulation using microbial model has been proved to be effective tool to study the in body process of drugs, especially drugs like ART which have rapid pharmacokinetics [42,43]. Microbial transformation could also provide important route to obtain novel bioactive agents. In our study, a facile microbial model was established for ART metabolism simulation.

Meanwhile, the UPLC-ESI-Q-TOF-MS^E^ method was successfully employed for identification of the metabolites of ART both in vivo and in vitro. And the combination analysis of microbial transformation of ART with the metabolism in vivo was performed for the first time. This rapid, efficient, and accurate identification method is efficient for screening strains from numerous fungi with ability to metabolize foreign organic substances [44,45]. And the microbial model may allow for identification and isolation of large quantities of metabolites, avoiding use of large numbers of animals. It was believed that in a manner similar to mammalian systems.

## 4. Materials and Methods

### 4.1. Materials and Reagents

ART and DHA were obtained from the Chongqing Wuling Mountain Pharmaceutical, Kunming Pharmaceutical Group (Chongqing, China, the batch number is MFCD00081057, C00220160402, respectively). The compound had purity ≥99%. De-ART was provided by Professor Wang M.Y. (Capital Medical University, Beijing, China). *Cunninghamella elegans* CICC 40250 was purchased from the China Center of Industrial Culture Collection (Beijing, China). Reagents used were high-performance liquid chromatography (HPLC) grade. Acetonitrile and methanol were purchased from Fisher (Fisher Chemical, Geel, Belgium), while formic acid and ethyl acetate were purchased from Beijing Chemical Works (Beijing, China). Water was prepared using a Milli-Q system operating at 18.2 MΩ (Millipore, Bedford, MA, USA). All other chemicals used were purchased from Fisher Scientific or Beijing Chemical Works, and were of the highest purity available.

### 4.2. Instruments and LC–MS/MS Conditions

The UPLC-ESI-Q-TOF-MS^E^ system consisted of a Waters ACQUITY I-class UPLC and Xevo G2-XS Q-TOF Mass Spectrometer (Waters, Manchester, UK), equipped with an electrospray ionization (ESI) source (Waters, Manchester, UK). Chromatographic separation was achieved using an Acquity UPLC BEH C_18_ (1.0 mm × 150 mm, 1.7 μm, Waters). The mobile phase consisted of solvent A (H_2_O containing 0.1% formic acid *v*/*v*) and solvent B (acetonitrile containing 0.1% formic acid *v*/*v*). The gradient program for plasma samples, which included five segments, was as follows, 5–100% B from 0 to 15 min, 100–5% B from 15 to 17 min, and a post-run of 3 min for column equilibration. The flow rate was 0.4 mL min^−1^ and the temperature was held at 25 °C throughout the period of analysis.

The MS was operated in positive ionization mode across a scan range of 50 to 1000 *m*/*z*, with a scan time of 0.2 s. Source parameters: source temperature 120 °C, cone gas 50 L/h, desolvation temperature 450 °C, and desolvation gas flow 800 L/h. Argon (99.95%) was used for collision induced dissociation, and N_2_ was used as the drift gas. The low collision energy was set to 6 eV, and the high collision energy was ramped from 12 to 25 eV. MS^E^ analysis was conducted using multiple reactions monitoring with positive-ion electrospray ionization. The MS was calibrated weekly by external calibration using the Waters major mix. Leucine enkephalin was used for lock mass correction with *m*/*z* 556.2771 at an interval of 0.5 min to ensure robust and accurate mass measurements.

### 4.3. MS Data Processing

All data was collected using MassLynx4.1 (Waters, Manchester, UK) and processing was performed in UNIFI 1.9 (Waters, Manchester, UK). The components were identified in a nontargeted manner by spectral deconvolution in UNIFI 1.9 (Waters, Manchester, UK) by the following 3D peak detection features; low-energy limits of 150 and high-energy limits of 20, isotope clustering, and high-to low energy association within a 0.5 fraction of the chromatographic and drift peak with a mass accuracy of ±2 mDa. The maximum number of allowed fragment ions per match was set at 10.

### 4.4. Animal Handling and Sample Preparation

Male C57 mice (180–200 g) were supplied by Beijing Fang Yuanyuan Farm (Grade II, Certificate No. SCXK 2014-0012). The experimental protocol was approved by the Ethics Committee and conformed to the ‘Principles of Laboratory Animal Care’. Mice were fasted for 12 h before drug administration and for a further 3 h after dosing. Water was freely available to the mice during the experiments. Mice were given a single oral dose of ART (100 mg/kg). ART was formulated as a suspension in corn oil. Blood was collected 3 h after administration by eye removal. Three hours was chosen based on a preliminary study showing that 2.5 h after oral administration of artemisinin, the blood drug concentration decreased rapidly and metabolites increased. The control sample was prepared identical to the test sample except for oral administration.

Plasma and red blood cells were separated by centrifugation (Fersco21, Thermo Fisher, Osterode, Germany) at 860× *g* for 15 min, at 4 °C. Plasma samples (n = 3) were loaded onto pretreated solid phase extraction cartridges (Oasis extraction cartridges, Waters, Milford, MA, USA). The cartridges were washed with 1 mL of water and analytes eluted with 1 mL of methanol. The methanol fractions were dried by Vacuum freeze concentrator (Xiangyi, Changsha, China). All the samples were reserved at −80 °C until reconstituted with 100 µL of initial mobile phase and analyzed immediately. Aliquots (10 µL) of the reconstituted solutions were injected onto the LC/MS.

### 4.5. Culture and Biotransformation Procedures

The following medium was used in the culture and bioconversion experiments; 20 g Sabouraud Dextrose Broth (Oxoid), 10 g peptone (Solarbio, Beijing, China), 15 g sucrose (Solarbio, Beijing, China), and 1000 mL deionized water. Cultures were grown on sterile plates.

Fungal mycelia from disks were transferred into 500 mL shaker flasks containing 100 mL of medium. After 48 h of incubation at 28 °C and 180 rpm on a rotary shaker, 10 mL cultures containing mycelia were transferred to inoculate 500 mL Erlenmeyer flasks containing 100 mL of medium. The inoculated flasks were incubated for 48 h on rotary shakers at 28 °C and 180 rpm before addition of ART. ART was dissolved in acetone at a concentration of 25 mg/mL, and 2 mL of the solution was added into each flask. A total of 50 mg of substrate was used in the biotransformation, and the final concentration of ART in the fermentation system was 0.5 mg/mL. The cultures were incubated under the same conditions for an additional 14 days.

When fermentation was complete, the mycelia and broth were separated by centrifugation (1180× *g*, 20 min) and the mycelia were discarded. Aliquots of 10 mL of the supernatant were extracted with an equal volume of ethyl acetate (EtOAc) three times. The organic phase was collected and evaporated under vacuum, resulting in a brown residue. The residues were dissolved in acetonitrile. The biotransformation sample was obtained after by centrifugation at 13,870× *g* for 5 min.

## 5. Conclusions

In conclusion, our results demonstrated the ability of *Cunninghamella elegans* CICC 40250 to mimic in vivo ART metabolism. This strain was able to produce most types of metabolites found in vivo. The ability of this model to mimic mammalian metabolism, to perform biotransformation reactions, and to produce significant amounts of drug metabolites, suggested that this microbial system represents a suitable alternative for drug metabolism studies. The suitability of this model may eliminate the need to use large quantities of animals in experimental research. Furthermore, ART metabolites were accurately and rapidly identified by UPLC-ESI-Q-TOF-MS^E^, and DHA was detected and identified as a metabolite of ART in mouse plasma for the first time. This method for screening suitable strains demonstrated the possibility of obtaining valuable metabolites in large quantities in vitro.

## Figures and Tables

**Table 1:** Summary of metabolites of ART detected in mouse plasma (in vivo) and microbial transformation system (in vitro).

No.	Component	Formula	Observed Retention Time (min)	Plasma (In Vivo)		Fermentation Broth (In Vitro)		Major Fragments
				Observed *m*/*z*	Mass Error (mDa)	Observed *m*/*z*	Mass Error (mDa)	
M1	ART + O	C_15_H_22_O_6_ + H^+^	2.6	299.1487	−0.2	-	-	321, 299, 337, 281, 263, 253, 235, 217, 207
M2	ART + O	C_15_H_22_O_6_ + Na^+^	2.8	321.1301	−0.5	321.1312	0.3	321, 337, 299, 281, 263, 253, 235, 221, 207, 189
M3	ART + O	C_15_H_22_O_6_ + Na^+^	2.9	321.1301	−0.4	321.1309	0.1	321, 337, 299, 281, 263, 245, 235, 221, 217, 189, 175
M4	ART + O	C_15_H_22_O_6_ + Na^+^	3.0	321.1304	−0.4	321.1305	−0.3	321, 337, 299, 283, 265, 235, 223, 219, 207, 189, 147
M5	ART + O	C_15_H_22_O_6_ + Na^+^	3.5	321.1307	−0.1	321.1304	−0.5	321, 337, 281, 263, 245, 235, 217, 205, 189
M6	ART + O	C_15_H_22_O_6_ + Na^+^	3.7	321.1304	−0.4	321.1302	1.8	321, 337, 299, 281, 263, 235, 221, 207, 193, 189, 175
M7	ART + O	C_15_H_22_O_6_ + Na^+^	3.9	321.1301	−0.7	-	-	321, 299, 337, 281, 253, 245, 235, 223, 217, 177, 149
M8	ART + O	C_15_H_22_O_6_ + H^+^	4.0	299.1481	−0.8	321.1301	−0.8	321, 337, 283, 263, 253, 235, 221, 207, 189, 175
M9	ART + O	C_15_H_22_O_6_ + Na^+^	4.2	-	-	321.1306	−0.3	321, 337, 307, 267, 253, 249, 235, 207, 189, 175, 165
M10	ART + O	C_15_H_22_O_6_ + Na^+^	4.7	-	-	321.1301	−0.7	321, 299, 337, 283, 261, 237, 235, 219, 205, 177, 165
M11	ART + O	C_15_H_22_O_6_ + Na^+^	4.9	337.1038	−0.6	337.1039	−0.9	321, 299, 337, 281, 253, 235, 207, 189, 179
M12	ART + O	C_15_H_22_O_6_ + Na^+^	5.1	-	-	321.1296	−1.3	321, 337, 299, 281, 263, 253, 235, 207, 189, 175, 149
M13	ART + O	C_15_H_22_O_6_ + Na^+^	5.8	-	-	321.1304	−0.4	321, 337, 299, 281, 263, 235, 217, 207, 189, 175, 149
M14	ART + O2	C_15_H_22_O_7_ + Na^+^	2.0	337.1254	−0.4	337.1275	1.8	337, 353, 315, 297, 279, 261, 251, 233, 205
M15	ART + O2	C_15_H_22_O_7_ + Na^+^	2.2	-	-	337.1246	−1.1	337, 353, 277, 255, 237, 219, 209, 191, 163, 135
M16	ART + O2	C_15_H_22_O_7_ + Na^+^	2.2	337.1255	−0.2	-	-	337, 315, 267, 252, 226, 199,176
M17	ART + O2	C_15_H_22_O_7_ + Na^+^	2.3	337.1259	0.1	-	-	337, 297, 279, 265, 247, 233, 217, 201, 189
M18	ART + O2	C_15_H_22_O_7_ + Na^+^	2.4	-	-	337.1256	−0.1	337, 353, 299, 299, 281, 277, 263, 255
M19	ART + O2	C_15_H_22_O_7_ + Na^+^	2.5	-	-	337.1247	−1.0	337, 353, 277, 253, 235, 219, 209, 207, 163, 135
M20	ART + O2	C_15_H_22_O_7_ + Na^+^	3.0	337.1274	1.7	-	-	337, 321, 299, 281, 279, 263, 252, 235, 207, 189, 147
M21	ART + O2	C_15_H_22_O_7_ + Na^+^	3.6	337.1281	−0.4	337.1068	−1.1	337, 353, 305, 297, 279, 233, 221, 177
M22	ART + O2	C_15_H_22_O_7_ + Na^+^	3.8			337.1244	−1.3	337, 353, 315, 297, 279, 261, 251, 233, 205, 191, 175
M23	de-ART	C_15_H_22_O_4_ + H^+^	8.3	267.1586	−0.5	267.1586	−0.5	289, 267, 305, 249, 239, 231, 221, 207, 203, 193, 179
M24	de-ART + O	C_15_H_22_O_5_ + Na^+^	4.5	-	-	305.1355	−0.5	305, 321, 283, 265, 247, 237, 219, 201, 189
M25	de-ART + O	C_15_H_22_O_5_ + H^+^	4.9	283.1536	−0.4	283.1535	−0.3	283, 305, 321, 265, 247, 239, 191
M26	de-ART + O	C_15_H_22_O_5_ + Na^+^	5.2	283.1531	−0.9	283.1355	−0.5	283, 305, 265, 247, 237, 219, 191, 177,
M27	de-ART + O	C_15_H_22_O_5_ + Na^+^	5.4	-	-	305.1354	−0.6	305, 321, 267, 247, 219, 205, 177, 159
M28	de-ART + O	C_15_H_22_O_5_ + Na^+^	5.6	305.1355	−0.4	305.1358	−0.1	283, 305, 321, 265, 247, 219, 205, 177, 159
M29	de-ART + O	C_15_H_22_O_5_ + Na^+^	5.8	305.1358	−0.1	-	-	305, 283, 276, 249, 239, 231, 221, 207, 203, 193, 179
M30	de-ART + O	C_15_H_22_O_5_ + Na^+^	6.0	305.1353	−0.8	305.1353	−0.6	305, 321, 265, 247, 219, 205, 193, 149
M31	de-ART + O	C_15_H_22_O_5_ + Na^+^	7.2	-	-	305.1355	−0.5	305, 321, 283, 247, 223, 195, 167
M32	DHA	C_15_H_24_O_5_ + Na^+^	7.0	307.1505	−1.1	-	-	307, 323, 267, 249, 239, 231, 221, 203, 163
M33	DHA + O	C_15_H_24_O_6_ + Na^+^	2.2	-	-	323.1456	−1.0	323, 339, 283, 265, 247, 219, 189
M34	DHA + O	C_15_H_24_O_6_ + Na^+^	2.5	-	-	323.1458	−0.7	323, 339, 283, 265, 247, 219, 191
M35	DHA + O	C_15_H_24_O_6_ + Na^+^	3.1	-	-	323.1458	−0.8	323, 339, 285, 267, 249, 219, 191, 177
M36	DHA + O	C_15_H_24_O_6_ + Na^+^	3.7	323.1449	−1.6	323.1484	1.8	323, 283, 265, 253, 247, 237, 219, 209
M37	DHA + O	C_15_H_24_O_6_ + Na^+^	3.9	-	-	323.1456	−1.0	323, 285, 267, 249, 239, 235, 231, 221, 203
M38	DHA-O	C_15_H_24_O_6_ + Na^+^	6.6	-	-	291.1561	−0.3	291, 307, 279, 269, 251, 233, 223, 215, 205, 179
M39	DHA-O + O	C_15_H_24_O_5_ + Na^+^	5.8	307.1513	−0.4	307.1512	−0.5	307, 323, 267, 249, 239, 231, 221, 203, 179

Observed *m*/*z* of the precursor ions were recorded at low collision energy, and the major fragment ions were recorded at high collision energy.
